# Distinct Breast Tissue Microbiota Profiles in Early-Stage Breast Cancer: A Prospective Study in Turkish Women

**DOI:** 10.3390/life15101518

**Published:** 2025-09-26

**Authors:** Mehmet Fatih Özsaray, Turgay Şimşek, Deniz Sünnetçi Akkoyunlu, Naci Çine, Nuh Zafer Cantürk

**Affiliations:** 1 Department of General Surgery, Kocaeli University, Kocaeli 41001, Turkey; f_ozsaray@hotmail.com (M.F.Ö.); genel.cerrah@hotmail.com (T.Ş.); 2 Department of Medical Genetics, Kocaeli University, Kocaeli 41001, Turkey; deniz.sunnetci@kocaeli.edu.tr (D.S.A.); naci.cine@kocaeli.edu.tr (N.Ç.)

**Keywords:** breast cancer tissue, normal breast tissue, gut sample, microbiota

## Abstract

**Background:** This pilot study aimed to investigate the relationship between the breast tissue microbiota and breast cancer in Turkish women. We compared cancerous and adjacent normal breast tissues, as well as stool samples, obtained during breast-conserving surgery. **Methods:** In this prospective study, paired tumor and normal breast tissue samples, together with preoperative stool samples, were analyzed using 16S rRNA sequencing. Diversity indices and relative abundance differences were calculated, with effect sizes, 95% confidence intervals, and false discovery rate (FDR) corrections reported where appropriate. **Results:** A total of 22 patients with early-stage breast cancer were included (mean age 58.3 ± 12.7 years, mean BMI 28.9 ± 3.1 kg/m^2^). Distinct compositional shifts were observed between tumor and normal tissues, with *Ruminococcus*, *Eubacterium*, *Actinobacteria* (*phylum*), and *Stenotrophomonas* enriched in tumor samples, while *Lactobacillus*, *Staphylococcus*, *Bifidobacterium*, and *Faecalibacterium* were more abundant in normal tissues. No consistent associations were identified between fecal and breast tissue microbiota. **Limitations:** The small sample size, absence of healthy tissue or stool controls, and reliance on 16S rRNA sequencing limit the generalizability and functional interpretation of these findings. **Conclusions:** Despite these limitations, this study demonstrates localized microbial differences between tumor and adjacent normal breast tissues. Larger, multi-center studies with healthy controls and functional omics approaches are warranted to clarify the biological relevance and potential clinical implications.

## 1. Introduction

Breast cancer is one of the most common malignancies among women worldwide and remains a major public health concern [1]. According to GLOBOCAN 2020 data, more than 2 million new cases were diagnosed globally, including over 24,000 women in Turkey [2]. The incidence of breast cancer continues to rise, and it is estimated that one in every 8–10 women will develop breast cancer during their lifetime [3].

Multiple factors, including age, hormonal influences, genetic predisposition, lifestyle, and environmental exposures, contribute to breast cancer risk and disease progression [1,3]. In addition to these well-recognized factors, the human microbiota has recently emerged as a potential player in cancer biology. The microbiota refers to the microbial communities inhabiting different sites of the human body, where they influence host immunity, metabolism, and hormone regulation [4,5]. Dysbiosis, or imbalances in microbial composition, has been implicated in several chronic diseases including obesity, diabetes, autoimmune disorders, and cancer [5].

Recent studies suggest that breast tissue harbors its own distinct microbiome, independent of the gut [6]. Moreover, multi-omics analyses have demonstrated compositional shifts between malignant and adjacent normal tissues, suggesting a potential role for microbial changes in carcinogenesis [7]. However, data from Turkish populations remain limited, despite potential differences driven by genetics, diet, and environment.

Therefore, the aim of this pilot study was to compare the microbiota profiles of tumor and adjacent normal breast tissues, as well as stool samples, from Turkish women with early-stage breast cancer, using a within-patient design to minimize interindividual variability.

## 2. Materials and Methods

### 2.1. Study Design and Patient Selection

This pilot study was conducted prospectively between 2022 and 2023 at a single tertiary care center in Turkey. Female patients aged 30–75 years with histologically confirmed stage I–II invasive breast cancer who were scheduled for breast-conserving surgery were screened. Inclusion criteria were: no systemic antibiotic or corticosteroid use within the previous three months, no autoimmune or chronic inflammatory diseases, no previous malignancy, and no pregnancy or lactation during the study period. Patients who declined participation or failed to provide stool samples were excluded.

### 2.2. Sample Collection and Storage

During surgery, tumor tissue and adjacent normal breast tissue (at least 2 cm from the tumor margin) were aseptically collected and immediately stored in RNase/DNase-free tubes at −80 °C. Fresh stool samples were obtained one day before surgery using sterile collection kits (Qiagen, Hilden, Germany) and stored at −80 °C until DNA extraction. To minimize contamination, negative controls (blank extractions and PCR controls) were included throughout DNA extraction and sequencing procedures. Only samples passing quality control thresholds (minimum sequencing depth of 20,000 reads after filtering) were retained for downstream analysis.

### 2.3. DNA Extraction and 16S rRNA Sequencing

Genomic DNA was extracted from tissue samples using the DNeasy Blood & Tissue Kit (Qiagen Hilden, Germany) and from fecal samples using the PureLink™ Microbiome DNA Purification Kit (Invitrogen Thermo Fisher Scientific, Waltham, MA, USA) according to manufacturer instructions. DNA concentration and quality were measured using a NanoDrop spectrophotometer (Thermo Scientific) and Qubit Fluorometer.

Libraries targeting variable regions V2, V3, V4, V6–7, V8, and V9 were prepared using the 16S Metagenomics Kit (Thermo Fisher Scientific). Sequencing was performed on an Ion S5™ XL platform, generating an average of 50,000–80,000 reads per sample.

### 2.4. Sequence Data Processing and Bioinformatics

**Figure 1:**
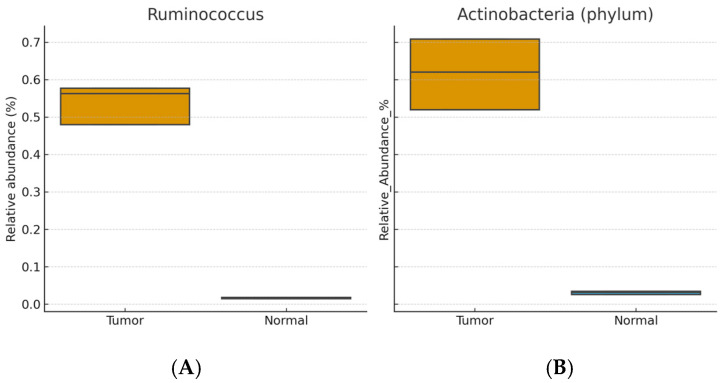
Relative abundance (%) of (**A**) *Ruminococcus* and (**B**) Actinobacteria (phylum) in paired tumor vs. adjacent normal breast tissue (n = 22). Boxes show median and interquartile range (IQR); whiskers denote 1.5 × IQR. Statistics: Wilcoxon signed-rank test with Benjamini–Hochberg FDR correction; adjusted *p* values are indicated above panels.

**Figure 2:**
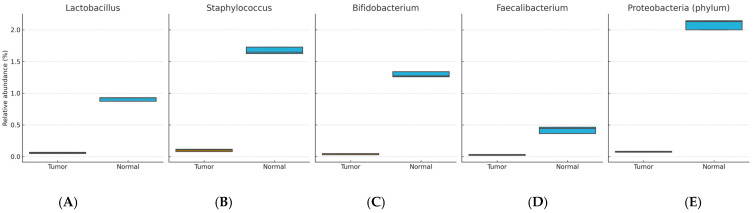
Relative abundance (%) of (**A**) *Lactobacillus*, (**B**) *Staphylococcus*, (**C**) *Bifidobacterium*, (**D**) *Faecalibacterium*, and (**E**) Proteobacteria (phylum) in paired tumor vs. normal tissues (n = 22). Box-plot details and statistics are as in Figure 1.

**Figure 3:**
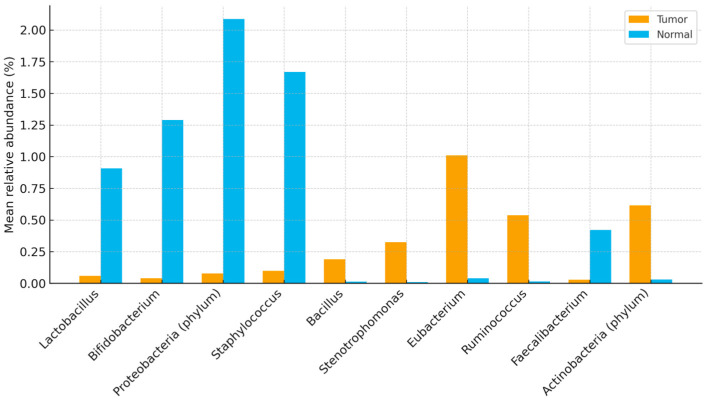
Differentially abundant genera between tumor and normal tissues, ordered by absolute effect size (|r|). Bars show mean relative abundance (%) ± SEM. Significance after Benjamini–Hochberg FDR correction is indicated by asterisks.

**Figure 4:**
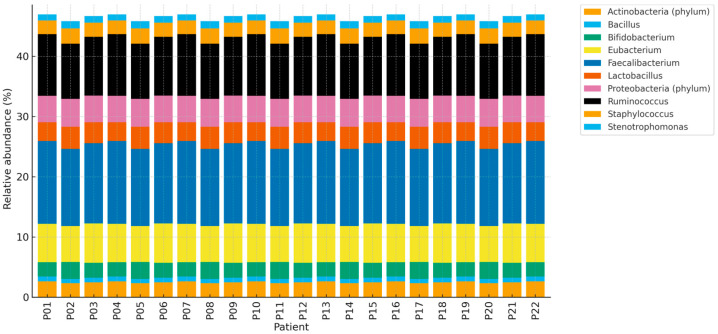
Genus-level relative abundance (%) profiles of fecal microbiota across 22 patients (stacked bars). Overall similarity is further shown in Supplementary Figure S1 (heatmap) and Supplementary Figure S2 (PCoA; Bray–Curtis dissimilarity).

Raw reads were processed using Ion Torrent Suite and analyzed in QIIME2 (v2021.8). Denoising was performed with DADA2 to obtain amplicon sequence variants (ASVs)**.** Taxonomic assignment used a naïve Bayes classifier trained on SILVA v138 (99% similarity). Data were rarefied to 20,000 reads per sample before diversity analyses. For compositional comparisons, abundances were converted to relative abundance (%); all figures (Figure 1, Figure 2, Figure 3 and Figure 4) use 0–100% Y-axes with units clearly labeled. Raw counts were not used for between-group visualization or inference.

### 2.5. Clinical and Dietary Data

Clinical and demographic variables including age, BMI, menopausal status, comorbidities, oral contraceptive use, family history of breast cancer, antibiotic use, and COVID-19 infection/vaccination history were recorded. Dietary habits were collected using a self-reported questionnaire; however, validated dietary assessment tools were not employed, which represents a limitation of the study.

### 2.6. Statistical Analysis

Statistical analyses were performed using IBM SPSS Statistics v20.0 (SPSS Inc., Chicago, IL, USA). Normality was tested using the Kolmogorov–Smirnov and Shapiro–Wilk tests. Normally distributed data are presented as mean ± standard deviation; non-normally distributed variables as median (interquartile range); categorical variables as frequency (%). Differences between groups were tested using independent sample t-test or Mann–Whitney U test, as appropriate. For paired comparisons, paired t-test or Wilcoxon signed-rank test was applied. Relationships between categorical variables were examined using Chi-square or Fisher’s exact test.

For microbial diversity analyses, PERMANOVA was used to test beta diversity differences. To strengthen interpretation, effect sizes (Cohen’s d or r) and 95% confidence intervals were calculated where appropriate. To control for multiple comparisons, *p*-values were adjusted using the Benjamini–Hochberg false discovery rate (FDR) method, unless otherwise specified. A two-tailed *p*-value < 0.05 was considered statistically significant. For paired tumor–normal comparisons, we used the Wilcoxon signed-rank test and report effect sizes (r) alongside Benjamini–Hochberg false discovery rate (FDR)–adjusted *p* values. Box plots display median and interquartile range (IQR) with whiskers at 1.5 × IQR.

## 3. Results

This study enrolled 22 female patients with an average age of 58.3 ± 12.7 years and a mean BMI of 28.9 ± 3.1 kg/m^2^. Of these patients, 7 (31.8%) had a BMI < 30 and 15 (68.2%) had a BMI ≥ 30.

### 3.1. Association Between Clinical Variables and Microbiota Composition

To assess whether clinical parameters influenced microbial diversity, subgroup and correlation analyses were performed:

No significant differences in alpha diversity (Shannon index) were observed between premenopausal and postmenopausal women (*p* = 0.291, Cohen’s d = 0.24, 95% CI: −0.18–0.61).

BMI categories (<30 vs. ≥30) did not significantly affect microbial richness or beta diversity (Bray–Curtis dissimilarity, PERMANOVA *p* = 0.384, R^2^ = 0.02).

Recent history of antibiotic use, oral contraceptive use, menopausal status, and comorbidities showed no statistically significant associations with genus-level relative abundances (all *p* > 0.05 after FDR correction).

### 3.2. Microbial Differences Between Tumor and Normal Breast Tissue

Comparative analysis of paired tumor and adjacent normal breast tissues revealed significant compositional differences.

Enriched in tumor tissue: *Ruminococcus*, *Eubacterium*, Actinobacteria (phylum), *Stenotrophomonas*, and *Bacillus* (Wilcoxon signed-rank, BH-FDR–adjusted *p* < 0.05; r ≈ 0.3–0.5).

Enriched in normal tissue: *Lactobacillus*, *Staphylococcus*, *Bifidobacterium*, *Propionibacterium*, *Lactococcus*, Proteobacteria (phylum), *Burkholderia*, *Faecalibacterium*, and *Pelomonas* (BH-FDR–adjusted *p* < 0.05).

Figure 1 and Figure 2 show distributions of relative abundance (%) for selected taxa in paired tumor and adjacent normal tissues (boxplots display the median and interquartile range [IQR]; whiskers = 1.5 × IQR). Benjamini–Hochberg FDR–adjusted *p* values are indicated above each panel. Figure 3 displays differentially abundant genera as bar plots ordered by absolute effect size (|r|); bars denote mean relative abundance (%) ± SEM.

### 3.3. Fecal Microbiota Analysis

Fecal microbiota profiles were analyzed in all 22 patients.

No significant correlations were found between fecal and breast tissue (tumor or normal) microbiota (all *p* > 0.05).

Intra-cohort fecal microbiota composition was generally similar across patients, likely reflecting reported dietary homogeneity.

A cohort-level visualization of sample-to-sample similarity is provided in Supplementary Figure S1, with ordination shown in Supplementary Figure S2.

### 3.4. Summary of Findings

Overall, breast tumor tissue displayed dysbiosis compared with paired normal tissue, characterized by enrichment of potentially pro-inflammatory taxa (*Ruminococcus*, *Eubacterium*, *Actinobacteria* (*phylum*), *Stenotrophomonas*) and depletion of genera with possible protective roles (*Lactobacillus*, *Bifidobacterium*, *Faecalibacterium*). These localized microbial alterations were not mirrored in fecal microbiota profiles, suggesting that dysbiosis may be tissue-specific in breast cancer. These findings were consistent across patients, although the small cohort size limited subgroup analyses.

## 4. Discussion

This study provides new insights into the role of the breast microbiome in cancer biology by comparing tumor tissue, adjacent normal breast tissue, and stool samples from Turkish women with early-stage breast cancer. The paired design minimized interindividual variability and allowed for a direct comparison of microbial composition within the same patients. Our findings demonstrated significant compositional differences between tumor and normal tissues, whereas stool microbiota profiles remained relatively homogeneous and showed no significant correlation with breast tissue microbiota. These observations highlight the potential importance of localized microbial changes in breast carcinogenesis.

### 4.1. Comparison with Previous Literature

Our analysis revealed enrichment of *Ruminococcus*, *Eubacterium*, *Actinobacteria* (*phylum*), and *Stenotrophomonas* in tumor tissues compared with adjacent normal tissues. Similar microbial signatures have been reported in other studies, although with some variation across populations. For example, Urbaniak et al. (2016) [8] and Hieken et al. (2016) [9] demonstrated distinct microbial communities in malignant breast tissue, with specific taxa enriched in tumor compared to benign or normal samples. Banerjee et al. (2018) [10] described unique microbiological signatures in triple-negative breast cancer, reinforcing the idea that breast tumors harbor distinct microbial ecosystems. Our findings are consistent with these observations, particularly regarding the enrichment of potentially pathogenic or pro-inflammatory taxa in tumor tissue.

The increased abundance of *Actinobacteria* (*phylum*) and *Stenotrophomonas* in tumor tissue is noteworthy. Both genera have been associated with chronic inflammation and opportunistic infections in other contexts, and their presence in tumor microenvironments may reflect local immune modulation. Prior research has suggested that chronic inflammation contributes to carcinogenesis by generating reactive oxygen species and promoting genomic instability [11]. The enrichment of Actinobacteria (phylum) and Stenotrophomonas in tumors may be consistent with a more pro-inflammatory microenvironment; however, causality cannot be inferred from this cross-sectional design. These microbial signatures might aid risk stratification in the future, pending validation in larger, multi-center cohorts.

Conversely, we observed higher relative abundances of *Lactobacillus*, *Bifidobacterium*, and *Faecalibacterium* in normal breast tissues. These taxa are widely recognized for their anti-inflammatory and immunomodulatory properties. For example, *Lactobacillus* and *Bifidobacterium* species have been shown to enhance epithelial barrier function, produce short-chain fatty acids (SCFAs), and regulate host immune responses [4,6]. *Faecalibacterium prausnitzii* in particular is considered a marker of gut health due to its ability to produce butyrate, which exerts anti-inflammatory effects [12]. The depletion of these potentially protective taxa in tumor tissues may suggest a loss of microbial mechanisms that normally help maintain tissue homeostasis and immune balance.

Recent multi-omics studies have provided further evidence for functional differences between tumor-associated and normal breast tissue microbiota. Meng, S et al. (2018) [13] reported significant compositional shifts and metabolic alterations in breast cancer tissues, implicating microbiota in key pathways related to hormone metabolism, immune modulation, and carcinogen biotransformation. Kim, H.E et al. (2021) [14] demonstrated distinct microbial signatures in breast cancer tissues from Korean women, highlighting potential population-specific differences driven by genetics, diet, and environment. These findings underscore the importance of conducting microbiome studies in diverse populations, such as the present Turkish cohort, to capture regional variation. A recent systematic review by Bose, C. et al. (2025) [15] also emphasized the need to integrate metagenomic and metabolomic approaches to elucidate the functional significance of these microbial alterations.

### 4.2. Biological and Clinical Implications

The presence of distinct microbial signatures in breast tumors suggests several potential biological and clinical implications. First, the enrichment of pro-inflammatory taxa and depletion of beneficial commensals may influence local immune responses, potentially contributing to tumor growth and progression. These microbial imbalances may alter cytokine profiles, disrupt epithelial integrity, and modulate estrogen metabolism, all of which have been implicated in breast cancer pathogenesis [5,6].

Second, microbial signatures may serve as potential biomarkers for breast cancer diagnosis or prognosis. If validated in larger studies, the consistent depletion of *Lactobacillus* and *Bifidobacterium* and enrichment of *Actinobacteria* (*phylum*) and *Stenotrophomonas* could help identify patients at risk for more aggressive disease. Multi-omics approaches that integrate microbiome data with transcriptomic or metabolomic profiles may further enhance biomarker discovery.

Third, microbial alterations may offer novel therapeutic opportunities. Modulation of the microbiome through probiotics, prebiotics, antibiotics, or dietary interventions has shown promise in other cancers and chronic diseases. While our results do not establish causality, they support the hypothesis that restoring beneficial taxa or reducing pro-inflammatory microbes may have therapeutic potential in breast cancer. However, such applications remain speculative and require rigorous validation. If validated, these microbial signatures could complement existing clinicopathological markers in early-stage breast cancer.

### 4.3. Fecal Microbiota Findings

Unlike breast tissue samples, fecal microbiota profiles showed limited variability among patients and no significant association with tumor or normal tissue microbiota. This contrasts with some reports suggesting potential communication between gut and breast microbiota via immune or endocrine pathways [6]. Several factors may explain these discrepancies. First, our cohort size was relatively small, limiting statistical power. Second, dietary habits were self-reported and relatively homogeneous, which may have reduced variability in gut microbiota. Third, the absence of healthy stool controls limited the interpretability of fecal findings. Together, these observations suggest that while the gut microbiota may influence systemic cancer risk factors, breast tissue dysbiosis appears to be primarily a localized phenomenon in our cohort.

### 4.4. Study Limitations

Several limitations of this study should be acknowledged. The sample size was modest (n = 22), limiting generalizability and statistical power to detect subtle associations. The cross-sectional design precludes causal inferences regarding whether microbial alterations are drivers or consequences of breast cancer. The lack of healthy breast tissue or stool controls restricts our ability to establish baseline comparisons. Dietary data were self-reported using non-validated questionnaires, which may have introduced bias. Methodologically, the use of 16S rRNA sequencing limited taxonomic resolution to the genus level and did not provide functional insights into microbial activity. Future studies employing shotgun metagenomics, metabolomics, or transcriptomics are required to clarify functional roles.

### 4.5. Future Directions

Future research should address these limitations and expand upon the current findings:

Larger, multi-center studies: To validate microbial signatures across diverse ethnic and geographic populations.

Longitudinal designs: To determine whether microbial shifts precede tumorigenesis or arise as a consequence of malignancy.

Functional analyses: Incorporating metagenomic, metabolomic, and transcriptomic approaches will be essential to identify metabolic pathways influenced by the microbiota.

Inclusion of healthy controls: Both tissue and stool samples from cancer-free individuals are necessary to establish reference baselines.

Dietary assessment: Standardized and validated dietary tools should be employed to minimize bias in gut–breast microbiota comparisons.

Therapeutic exploration: While speculative, interventions aimed at modulating the breast microbiome—such as probiotics, prebiotics, or dietary modifications—should be evaluated for potential preventive or adjunctive roles in breast cancer management.

## 5. Conclusions

This pilot study compared the microbial profiles of tumor tissue, adjacent normal breast tissue, and stool samples in Turkish women with early-stage breast cancer. We observed significant differences between tumor and normal tissues, with enrichment of potentially pro-inflammatory taxa such as *Ruminococcus*, *Eubacterium*, *Actinobacteria* (*phylum*), and *Stenotrophomonas* in tumors, and depletion of potentially protective genera including *Lactobacillus*, *Bifidobacterium*, and *Faecalibacterium*. In contrast, stool microbiota profiles were relatively homogeneous and showed no significant correlation with breast tissue composition, suggesting that dysbiosis in breast cancer is a localized rather than systemic phenomenon.

Although limited by small sample size, lack of healthy controls, and reliance on 16S rRNA sequencing, our study provides evidence that microbial alterations in breast tissue may be associated with tumor biology. These findings highlight the need for larger, multi-omics studies to clarify the functional roles of the breast microbiome and its potential value as a biomarker or therapeutic target in breast cancer.

## Data Availability

Due to ethics/consent restrictions, raw sequence data cannot be publicly shared. De-identified processed data (feature table, taxonomy, metadata) and the raw reads are available from the corresponding author upon reasonable request, subject to IRB approval.

## Supplementary Materials

The following supporting information can be downloaded at: https://www.mdpi.com/article/10.3390/life15101518/s1, Figure S1: Heatmap of fecal microbiota (patients × genera) showing relative abundance (%); Figure S2: PCoA (Bray–Curtis) of fecal microbiota with axes denoting the percentage of variance explained.

## Abbreviations

BMI	Body Mass Index

## References

[1] Harbeck N., Gnant M. (2017). Breast cancer. Lancet.

[2] Sung H., Ferlay J., Siegel R.L., Laversanne M., Soerjomataram I., Jemal A., Bray F. (2021). Global Cancer Statistics 2020: GLOBOCAN Estimates of Incidence and Mortality Worldwide for 36 Cancers in 185 Countries. CA Cancer J. Clin..

[3] Soerjomataram I., Bray F. (2021). Planning for tomorrow: Global cancer incidence and the role of prevention 2020–2070. Nat. Rev. Clin. Oncol..

[4] Fernández M.F., Reina-Pérez I., Astorga J.M., Rodríguez-Carrillo A., Plaza-Díaz J., Fontana L. (2018). Breast Cancer and Its Relationship with the Microbiota. Int. J. Environ. Res. Public Health.

[5] Eslami-S Z., Majidzadeh-A K., Halvaei S., Babapirali F., Esmaeili R. (2020). Microbiome and Breast Cancer: New Role for an Ancient Population. Front. Oncol..

[6] Song X., Wei C., Li X. (2022). The Relationship Between Microbial Community and Breast Cancer. Front. Cell. Infect. Microbiol..

[7] Parida S., Siddharth S., Xia Y., Sharma D. (2023). Concomitant analyses of intratumoral microbiota and genomic features reveal distinct racial differences in breast cancer. Breast Cancer.

[8] Urbaniak C., Gloor G.B., Brackstone M., Scott L., Tangney M., Reid G. (2016). The microbiota of breast tissue and its association with breast cancer. Appl. Environ. Microbiol..

[9] Hieken T.J., Chen J., Hoskin T.L., Walther-Antonio M., Johnson S., Ramaker S., Yao J.Z. (2016). The microbiome of aseptically collected human breast tissue in benign and malignant disease. Sci. Rep..

[10] Banerjee S., Tian T., Wei Z., Shih N., Feldman M.D., Peck K.N., Robertson E.S. (2018). Distinct microbiological signatures associated with triple negative breast cancer. Sci. Rep..

[11] Parida S., Sharma D. (2019). The power of small changes: Comprehensive analyses of microbial dysbiosis in breast cancer. Biochim. Biophys. Acta Rev. Cancer.

[12] Maioli T.U., Borras-Nogues E., Torres L., Barbosa S.C., Martins V.D., Langella P., Azevedo V.A., Chatel J.M. (2021). Possible Benefits of Faecalibacterium prausnitzii for Human Health: From Probiotic to Preventive Therapy. Front. Pharmacol..

[13] Meng S., Chen B., Yang J., Wang J., Zhu D., Meng Q., Sun Y. (2018). Study on the correlation between intestinal flora and breast cancer. Microb. Pathog..

[14] Kim H.E., Kim J.J., Maeng S.J., Oh B.J., Hwang K.T., Kim B.S. (2021). Microbiota of Breast Tissue and Its Potential Association with Regional Recurrence of Breast Cancer in Korean Women. J. Microbiol. Biotechnol..

[15] Bose C., Uczkowski N.G., Sukla K., Raval N., Haque M.M., Zhang Y., Varma B., Satagopan J.M. (2025). Breast cancer and microbiome: A systematic review highlighting challenges for clinical translation. BMC Women’s Health.

