# Supplementary figures and images for: Distinct Breast Tissue Microbiota Profiles in Early-Stage Breast Cancer: A Prospective Study in Turkish Women
Source: Life (Basel). 2025 Sep 26;15(10):1518. doi: 10.3390/life15101518 (PMC12565134; doi:10.3390/life15101518)

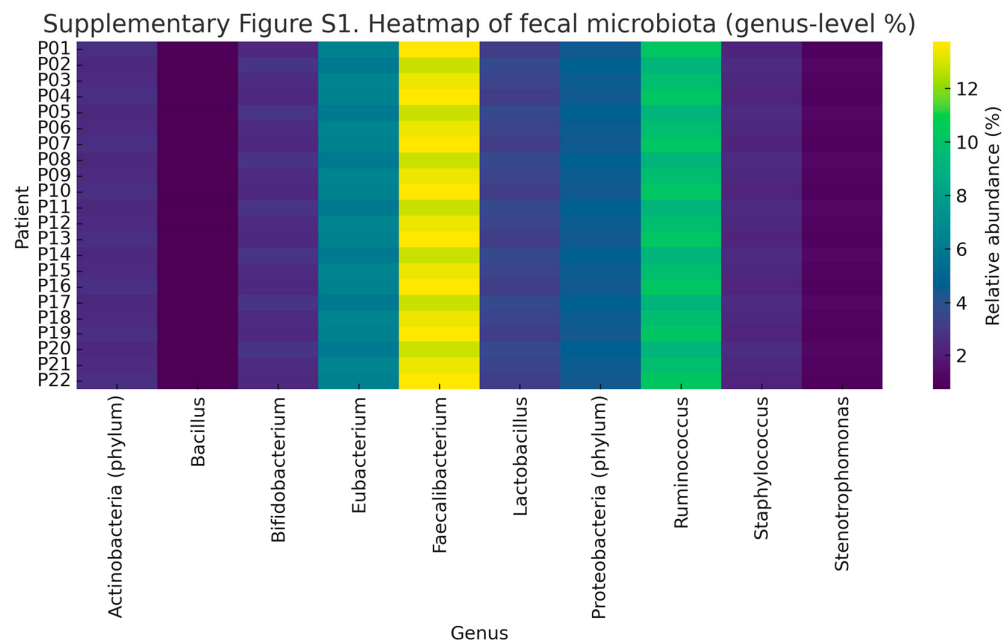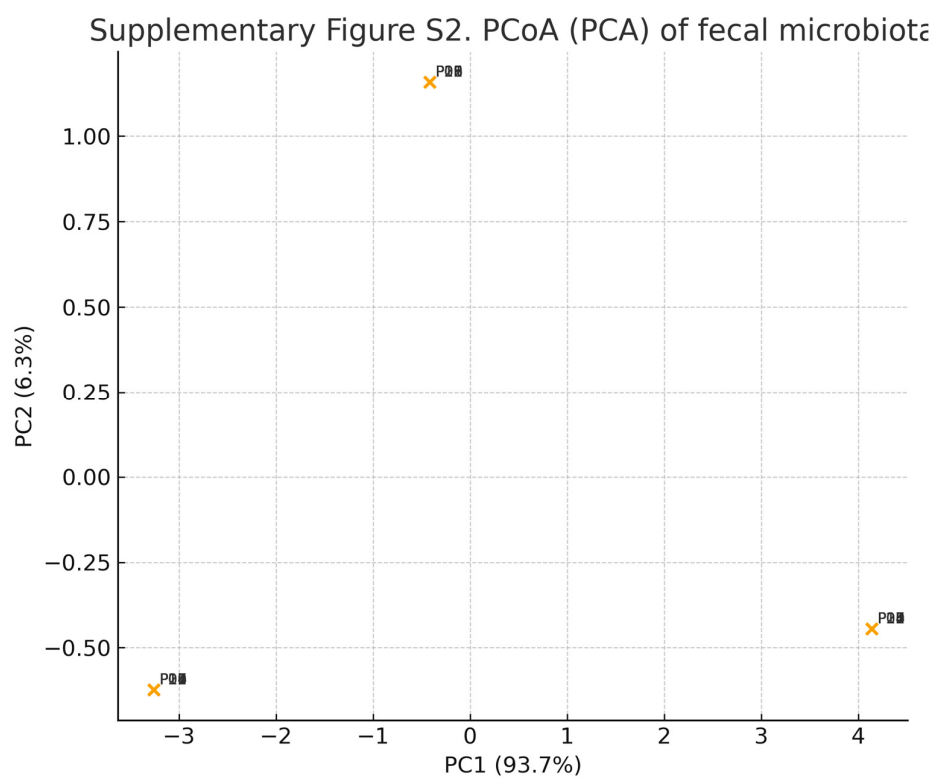

Supplement: Supplementary file 1 [file life-15-01518-s001.zip › life-3834121-supplementary.pdf]
